# A Novel Radiographic Measurement Method for the Evaluation of Metatarsophalangeal Joint Dislocation of the Lesser Toe in Patients with Rheumatoid Arthritis

**DOI:** 10.3390/ijerph18147520

**Published:** 2021-07-15

**Authors:** Hideki Ohashi, Keiichiro Nishida, Yoshihisa Nasu, Kenta Saiga, Ryuichi Nakahara, Masahiro Horita, Shunji Okita, Toshifumi Ozaki

**Affiliations:** 1 Department of Orthopaedic Surgery, Takahashi Central Hospital, Okayama 716-0033, Japan; hideki.kangeki0084@gmail.com; 2 Department of Orthopaedic Surgery, Okayama University Graduate School of Medicine, Dentistry and Pharmaceutical Sciences, Okayama 700-8558, Japan; masahiro.horita@gmail.com (M.H.); tozaki@md.okayama-u.ac.jp (T.O.); 3 Department of Orthopaedic Surgery, Okayama University Hospital, Okayama 700-8558, Japan; nasu_y@flute.ocn.ne.jp (Y.N.); ksaiga7@gmail.com (K.S.); pikumin55@gmail.com (R.N.); 4 Department of Orthopaedic Surgery, Okayama City Hospital, Okayama 700-0962, Japan; shunjiokita710@gmail.com

**Keywords:** lesser toe, metatarsophalangeal joint, rheumatoid arthritis, radiographic measurement, grading system

## Abstract

Dorsal dislocation of metatarsophalangeal (MTP) joints of the lesser toe frequently occurs in patients with rheumatoid arthritis (RA), and may cause painful and uncomfortable plantar callosities and ulceration. The current study examined the reliability and clinical relevance of a novel radiographic parameter (the MTP overlap distance [MOD]) in evaluating the severity of MTP joint dislocation. The subjects of the current study were 147 RA patients (276 feet; 1104 toes). MOD, defined as the overlap distance of the metatarsal head and the proximal end of the phalanx, was measured on plain radiographs. The relationship between the MOD and clinical complaints (forefoot pain and/or callosity formation) was analyzed to create a severity grading system. As a result, toes with callosities had a significantly larger MOD. ROC analysis revealed that the MOD had a high AUC for predicting an asymptomatic foot (−0.70) and callosities (0.89). MOD grades were defined as follows: grade 1, 0 ≤ MOD < 5 mm; grade 2, 5 ≤ MOD < 10 mm; and grade 3, MOD ≥ 10 mm. The intra- and inter-observer reliability of the MOD grade had high reproducibility. Furthermore, the MOD and MOD grade improved significantly after joint-preserving surgeries for lesser toe deformities. Our results suggest that MOD and MOD grade might be useful tools for the evaluation of deformities of the lesser toe and the effect of surgical intervention for MTP joints in patients with RA.

## 1. Introduction

The treatment of rheumatoid arthritis (RA) has made great progress in recent years with the widespread use of effective drugs that inhibit joint destruction, such as methotrexate and biologic disease-modifying antirheumatic drugs (bDMARDs). Young et al. [1] reported the prevalence of RA patients in the population who underwent joint replacement surgeries in the United States between 2002 and 2012. They showed that the prevalence among cases of elbow, shoulder, and ankle replacement surgeries decreased by 50%, 18%, and 38%, respectively. Cordtz et al. [2] evaluated the incidence of joint replacement before (1996–2001) and after (2003–2012) the introduction of bDMARDs in patients diagnosed with RA in Denmark between 1996 and 2012. They reported that the incidence of joint replacement in RA patients was stable from 1996 to 2001 but started to decrease from 2003, suggesting an association between the introduction of bDMARDs and a lower need for joint replacements among patients with RA. However, a significant population of patients still suffering from treatment failure even today due to a lack of efficacy, intolerance of DAMRDs, economic problems, or social backgrounds. This situation established the new concept “difficult to treat RA (DTRA)” [3,4], and functional reconstruction by surgery [5,6] as an essential non-pharmacological treatment option for DTRA.

In Japan, methotrexate was covered by insurance in 1999 for RA and the first bDAMRD was eligible for reimbursement in 2003. The IORRA cohort [7], one of the largest registries in Japan, reported an improvement in disease activity, a decreasing number of surgeries with the widespread use of bDMARDs, and an increasing trend in arthroplasties. Similarly, the largest multicenter registry of RA in Japan, the Ninja cohort [8], also reported that the disease activity of RA patients has dramatically improved since the 2000s, and the number of patients undergoing joint replacement surgery has been decreasing. In contrast, the number of patients undergoing hand and foot surgery has not yet decreased. A decreasing trend in disease activity among patients who underwent orthopedic surgeries was also reported [8]. Based on these trends, it can be inferred that the strategies of surgical treatment for RA may be changing due to advances in drug therapy.

One such change is the increasing demand for surgeries without sacrificing joint structures. Joint-preserving procedures are most commonly used for RA forefoot deformities. Foot joints, including the metatarsophalangeal (MTP) joint, are frequently affected sites in patients with RA, as well as the wrist and finger joints. Therefore, a significant population of patients with early RA already have affected joints in their feet. A significant problem involving RA forefoot deformities is plantar callosities associated with lesser toe deformities [9]. The dorsiflexion deformity and dorsal dislocation of the MTP joint are the greatest risk factors for plantar callosity formation [10]. Plantar callosities of the MTP joint often cause metatarsalgia requiring surgical intervention. To correct the malalignment or dislocation of the MTP joints, shortening of metatarsal bones is required to release soft tissue contractures. As an alternative to conventional MTP joint resection arthroplasty, a variety of shortening osteotomies for metatarsal bones have been performed without sacrificing the MTP joint, and good clinical outcomes have been reported [11,12,13,14,15,16,17,18,19]. Of note, there is no standardized radiographic measurement method to evaluate MTP joint deformities or the outcomes of these surgeries.

The objective of this study was to establish a practical grading method for MTP joint deformities based on quantitative radiographic measurements. Moreover, we sought to validate the reliability of the assessment for joint-preserving surgery of lesser toes in patients with RA.

## 2. Materials and Methods

### 2.1. Study Design

This retrospective study was conducted from April 2018 to March 2021 after receiving approval from the Institutional Review Board of Okayama University (no. 2194). Informed consent was obtained from all patients. We retrospectively reviewed the medical records of 280 RA patients admitted to our department for surgical treatment from July 2014 to December 2017. Inclusion criteria were: fulfilling the American Rheumatism Association criteria [20] or the American College of Rheumatology and the European League Against Rheumatism classification criteria [21], and the minimum age of 20 years. Exclusion criteria were: concomitant other connective tissue diseases, previous surgery on the ipsilateral ankle or foot, missing any data (weight-bearing radiographs, photographs of feet, or clinical scores at baseline and final visit), or less than six months follow-up periods.

### 2.2. Radiographic Measurement of Toe Deformities by the Mtp Overlap Distance (MOD)

**Figure 1:**
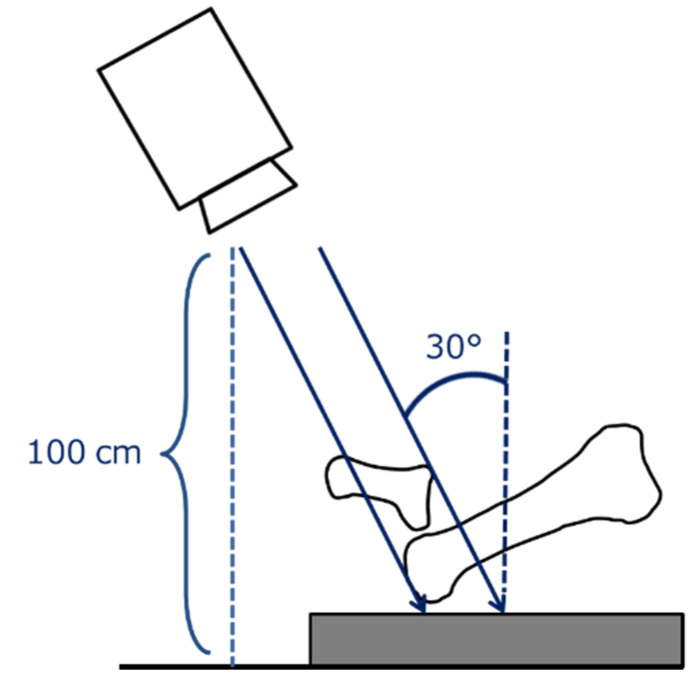
Method of plain radiography. Irradiation was performed from 100 cm and tilted forward by 30° from the vertical direction.

The weight-bearing dorsoplantar views of the foot were examined. Radiographs were obtained with the subjects in the standing position with the knees in full extension and the medial borders of the feet together. Irradiation was then performed from a distance of 100 cm and tilted forward by 30° from the vertical direction (Figure 1).

**Figure 2:**
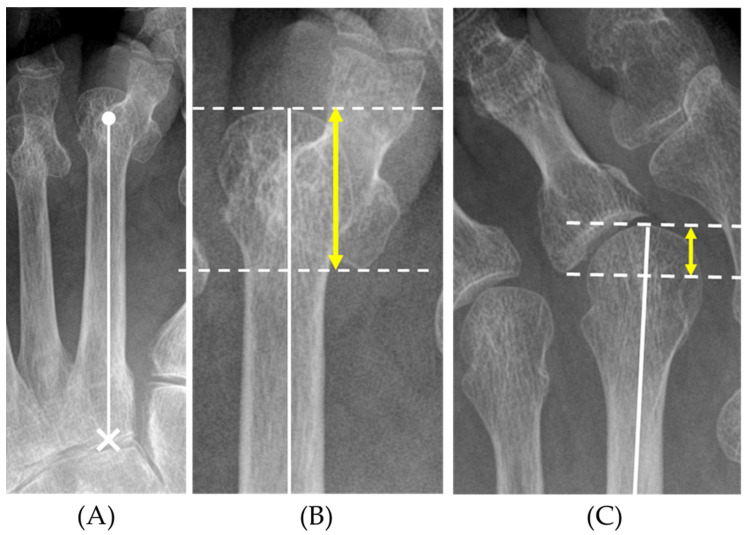
Definition of the MOD on plain radiographs. The metatarsal axis was defined as the line through the center of the metatarsal head (●) and the center of the proximal joint surface (×) (**A**). The MOD (↔) was defined as the distance between the tip of the metatarsal head and the most proximal point of the base of the proximal phalanx along the metatarsal axis (**B**). The MOD also reflects varus or valgus deformity to some extent (**C**). MOD: metatarsophalangeal overlap distance.

The degree of MTP joint deformity was assessed by the overlap of the metatarsal bone and proximal phalanx. We designated the distance between the tip of the metatarsal head and the most proximal point of the base of the proximal phalanx along the metatarsal axis as the MTP overlap distance (MOD). The metatarsal axis was defined as the line through the center of the metatarsal head and the center of the proximal joint surface [22]. The MOD also reflects varus or valgus deformity to some extent (Figure 2). The foot measurement was executed by H.O., S.O., and Y.N.

### 2.3. Grading of Forefoot Deformities Based on the MOD

The presence of plantar callosities was determined by recorded photographs. We then evaluated the association between the MOD of each toe and plantar callosities. We also assessed the MOD of the feet of asymptomatic RA patients. We created the MTP joint deformity grade (MOD grade) based on the Receiver Operating Characteristic (ROC) analysis of the MOD predicting toes of the foot without any symptoms (pain or callosities) and toes with callosities.

### 2.4. Investigation of Reproducibility

The inter- and intra-observer reliability were assessed using the intraclass correlation coefficient (ICC (2, 1) and ICC (1, 1), respectively). Three orthopedic surgeons (H.O., S.O., and Y.N. with 5, 8, and 14 years of experience, respectively) measured the MOD of 1104 target toes according to the established grading system. All 3 surgeons repeated these tests after 6 months to calculate the intra-observer reliability score.

### 2.5. Perioperative Assessment

To confirm the validity of the MOD and MOD grade as a tool for evaluating the surgical treatment of forefoot deformities, we analyzed the results of a shortening oblique osteotomy (SOO) using the MOD and MOD grade. Twenty-three RA patients (29 feet; 116 toes) who underwent SOOs of the metatarsal bones on all lesser toes with a minimum follow-up duration of 6 months were included in this assessment. The mean duration of follow-up was 19.3 ± 10.4 months. The operative procedure was reported by Nishida et al. [15]. In brief, an oblique osteotomy at the distal metaphysis of the metatarsal bone was performed. Then, the metatarsal length was shortened by sliding the metatarsal head fragment in the proximal and dorsal directions. The osteotomy site was fixed with a small bone screw. Resection of the minimum length of the metatarsal shaft to obtain adequate soft tissue tension was added, if necessary. Extensor digitorum brevis tendons were resected and the extensor digitorum longus tendons were extended in most cases. During this procedure, accurate sliding and shortening were achieved with internal fixation of the osteotomy site, which contributed to making a functional lateral arch. Furthermore, MTP joint motion was permitted postoperatively without wire fixation of any joints. For concomitant severe hallux valgus, a proximal first metatarsal bone osteotomy (14 feet) or arthrodesis of the first MTP joint (seven feet) was performed.

The pre- and post-operative clinical evaluations included the assessment of the Japanese Society of Surgery of the Foot (JSSF) score [23,24] and the Self-administered Foot Evaluation Questionnaire (SAFE-Q) [25]. The JSSF score consists of a standard rating system for the RA foot and ankle scale for pain (0–30 points), deformity (0–25 points), range of motion [ROM] (0–15 points), walking ability (0–20 points), and activities of daily living [ADL] (0–10 points). The SAFE-Q consists of 34 questionnaire items that provide five subscale scores, as follows: Pain and Pain-related; Physical Functioning and Daily Living; Social Functioning; Shoe-related; and General Health and Well-being. Clinical scores were collected by H.O., Y.N., K.S., R.N., M.H., S.O., and K.N.

Changes in the MOD, MOD grade, and the length of metatarsal bones were assessed on the preoperative plain radiographs and the final observation. The difference in magnification was corrected with the longitudinal length of the second cuneiform bone. We also checked plantar callosities at the final observation to compare the postoperative MOD and the metatarsal shortening length (as a percentage of the preoperative MOD) between the toes with and without callosities. We also examined the correlation between the pre- and post-operative changes (Δ) in clinical scores (JSSF-RA and SAFE-Q subscales) and the pre- and post-operative changes in the MOD (total from the second to fifth toes).

### 2.6. Statistical Analysis

The Student’s *t*-test, chi-square test, the Mann-Whitney *U* test, Tukey’s multiple comparison test, and Pearson’s correlation coefficient were used for statistical analyses in the present study. Statistical analysis was performed using Prism7 (GraphPad Software, San Diego, CA, USA) and SPSS (version 20; IBM Corp., Armonk, NY, USA). *p* values < 0.05 were considered statistically significant. Statistical analysis was performed by H.O. and Y.N.

## 3. Results

### 3.1. Measurement of MOD

Among the 280 patients, 147 (276 feet; 1104 lesser toes) were enrolled in this study. The mean age of subjects was 61.9 ± 13.2 years. The mean disease duration was 21.8 ± 10.2 years, and the mean Disease Activity Score 28 (DAS28)-CRP at admission was 2.56 ± 0.87. The specific operative procedures were as follows: 56 surgeries involving the hand or finger joints; 55 surgeries involving the ankles or feet; and 34 surgeries involving the shoulders or elbows. One patient underwent surgery on the spine and hip joint. Among the 276 feet studied, the overall mean value of the MOD was 5.3 ± 5.9 mm. The 3rd toe had the highest MOD. The MODs of the 2nd–5th toes were 5.7 ± 7.0, 6.1 ± 6.6, 5.0 ± 5.9, and 4.5 ± 3.2 mm, respectively (mean ± SD).

### 3.2. Grading of the MOD

**Figure 3:**
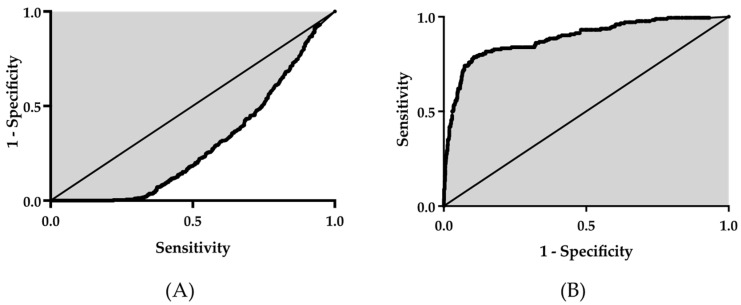
The ROC curve for predicting an (**A**) asymptomatic foot and (**B**) callosities by the MOD. The AUC was −0.70 (95% CI [−0.67, −0.73]) for an asymptomatic foot and 0.89 (95% CI [0.86–0.92]) for the prediction of callosities. ROC: receiver operating characteristic. AUC: area under the curve.

Sixty patients (96 feet) did not complain of forefoot pain or callosities. We identified MTP joint plantar callosities in 174 of 1104 toes. Most plantar callosities were observed in MTP joints of the second and third toes. The distribution of plantar callosities was as follows: 63 s toes; 66 third toes; 20 fourth toes; and 25 fifth toes. A comparison of the mean MOD values in the toe group with (*n* = 174) and without callosities (*n* = 930) showed that the callosities group had a significantly larger mean MOD (14.0 ± 7.3 mm vs. 3.7 ± 3.8 mm, *p* < 0.001; Table 1). ROC analysis indicated that the ROC area under the curve (AUC) was 0.89, the 95% CI was 0.86–0.92, and the optimal cut-off value was 7.8 mm (Table 1, Figure 3).

**Figure 4:**
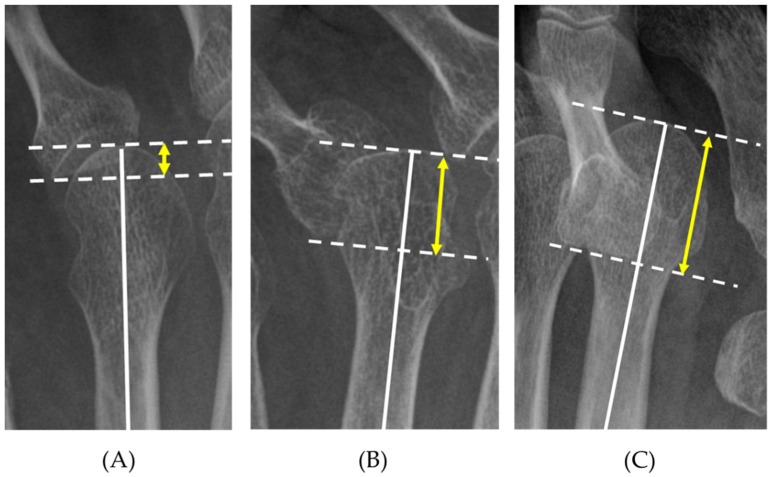
MOD grade. grade 1, 0 ≤ MOD < 5 mm (**A**), grade 2, 5 mm ≤ MOD < 10 mm (**B**), and grade 3, MOD ≥ 10 mm (**C**).

Similarly, the MOD had a significant AUC (−0.70, 95% CI [−0.67, −0.73]) for predicting asymptomatic toes (neither pain nor callosities). The MOD AUC also differed for each toe (−0.76, −0.70, −0.66, and −0.70 for the 2nd–5th toes, respectively). The calculated optimal cut-off value for the asymptomatic foot was 6.4 mm (2.0, 7.4, 4.5, and 4.3 mm for the 2nd to 5th toes, respectively). In addition to these calculated cut-off values, clinical practicability should be considered. We defined the MOD grade as follows: grade 1, MOD < 5 mm; grade 2, 5 ≤ MOD < 10 mm; and grade 3, MOD ≥ 10 mm (Figure 4). When the MOD cut-off value for plantar callosity was set to 10 mm, the sensitivity was 0.74, the specificity was 0.93, the positive predictive value was 0.65, and the negative predictive value was 0.95. To predict asymptomatic foot (pain or callosities) using the MOD with a cut-off value of 5 mm, the sensitivity was 0.90, the specificity was 0.41, the positive predictive value was 0.45, and the negative predictive value was 0.89. The rate of toes with plantar callosities in each MOD grade was significantly different by re-assessment of the 1104 toes, as follows: grade 1, 32/722 toes (4.1%); grade 2, 17/135 toes (12.6%); and grade 3, 125/197 toes (63.5%).

### 3.3. Investigation of Reproducibility

Based on the results of the reliability test by three independent observers, the intra-observer reliability of the MOD grade was 0.98 at ICC (1, 1) [95%CI: 0.98–0.99] and the inter-observer reliability was 0.96 at ICC (2, 1) [95% CI: 0.95–0.97], indicating an extremely high degree of reproducibility.

### 3.4. Perioperative Assessment

#### 3.4.1. Clinical Assessment

The mean JSSF total score of the final observation improved significantly from 62.4 ± 7.5 to 85.0 ± 9.4 points (*p* < 0.001). The mean SAFE-Q scores of the last observation also improved significantly from the preoperative observation (Pain-related, 59.3 ± 17.1 to 68.2 ± 14.5; Physical Functioning and Daily Living, 65.6 ± 17.2 to 71.0 ± 14.8; Social Functioning and Daily Living, 70.0 ± 23.1 to 76.6 ± 17.5; Shoe-related, 44.3 ± 21.3 to 51.9 ± 27.0; General Health and Well-being, 65.7 ± 24.5 to 75.2 ± 23.2; Table 2).

#### 3.4.2. Radiographic Assessment

**Figure 5:**
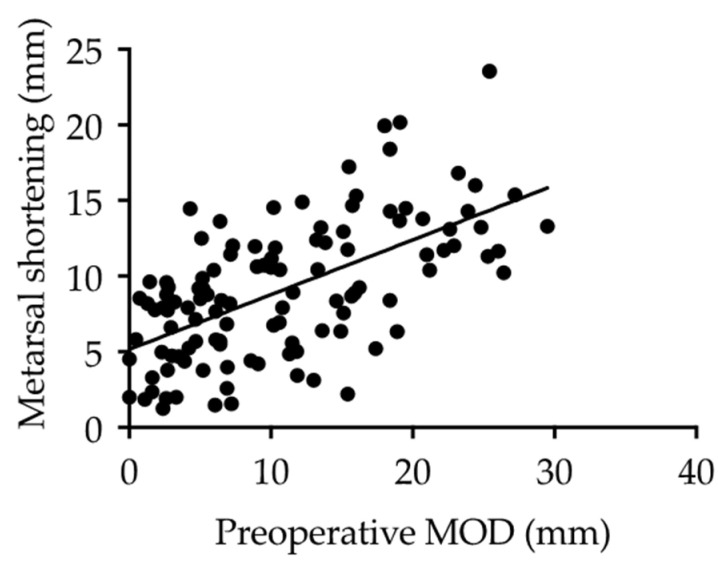
Correlation between the preoperative MOD and amount of metatarsal shortening. These data suggest there was a highly positive correlation between the preoperative MOD and metatarsal shortening (Pearson’s correlation, r = 0.60, *p* < 0.001).

**Figure 6:**
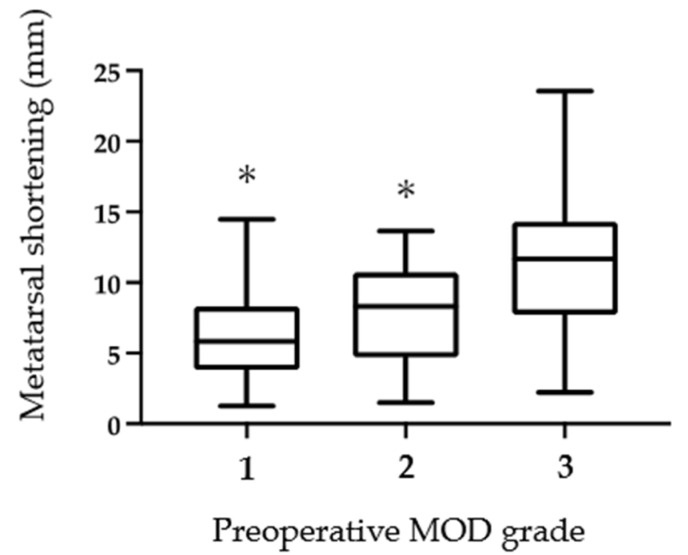
Shortening length of metatarsal bones in each preoperative MOD grade. The mean shortened length of metatarsal bones was significantly smaller in cases with grades 1 and 2 than in grade 3 (Tukey’s multiple comparisons test * *p* < 0.0001).

**Figure 7:**
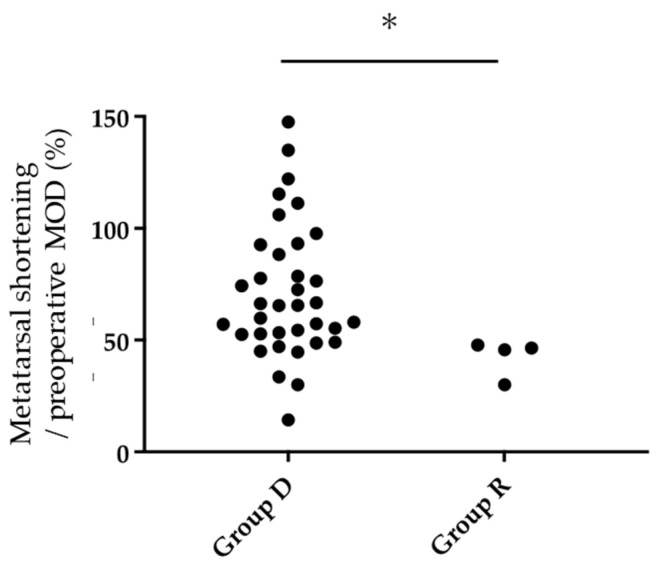
Assessment of metatarsal shortening length relative to the preoperative MOD. The ratio of metatarsal shortening length to the preoperative MOD (%) was significantly less in Group R with remnant callosities than in Group D without remnant callosities (Mann-Whitney U test, * *p* < 0.05) and <50% for all four toes.

The mean MOD of the final observation was significantly improved from the preoperative value (10.7 ± 7.5 to 4.8 ± 2.4 mm; *p* < 0.01). Furthermore, the MOD grade also improved significantly (Table 2). There was a significant positive correlation (r = 0.60, *p* < 0.01) between the shortening length of the metatarsal bones and the preoperative MOD value, suggesting that the larger preoperative MOD, the larger the amount of metatarsal shortening required by osteotomy to correct the MTP joint deformity (Figure 5). Similarly, as the preoperative MOD grade increased, the shortening length of the metatarsal bones increased significantly (Figure 6). In this study subset, plantar callosities were identified in 40 of 116 toes preoperatively. The plantar callosities disappeared in 36 toes (Group D), and four toes had remnant plantar callosities (Group R) at the final observation. The metatarsal shortening length-to-preoperative MOD ratio was significantly higher in Group D than in Group R (73.2 ± 29.2% vs. 42.5 ± 8.4%; *p* < 0.05). All four toes in Group R had a metatarsal shortening length-to-preoperative MOD ratio < 50% (Figure 7).

The Δ MOD (sum of the 2nd–5th toes) was only correlated with the JSSF subscale, deformity, and did not correlate significantly with other subscale scores (Table 3).

## 4. Discussion

### 4.1. Joint Preservation Surgeries for Rheumatoid Forefoot Deformities

Various modifications of joint-preserving procedures to correct RA-associated forefoot deformities have been performed. In our previous study [15], the SOO procedure showed satisfactory short-term clinical results [14] without fixation of the MTP joint and the phalangeal joints. Early postoperative range of motion (ROM) exercises for the MTP joint with reduced invasiveness are expected to improve postoperative ROM and function. As the osteotomy site of the metatarsal bone was rigidly fixed with bone screws, the amount of shortening was controlled, which enabled accurate planning of the postoperative forefoot alignment. Ebina et al. [11,12] reported that joint-preserving arthroplasty results in higher plantar pressures of the 1st MTP joint when compared to joint resection or replacement arthroplasty. In contrast, the plantar pressures of the 2nd and 3rd MTP joints are lower than metatarsal head resection procedures and these distributions are associated with better patient-based outcomes. Fukushi et al. [17] reported that joint-preserving procedures for rheumatoid forefoot deformities resulted in better clinical outcomes than resection arthroplasty with respect to hallux function and lesser toe alignment. With the advances in drug therapy, the application of these joint-preserving surgical procedures for forefoot deformity in RA has become more widely adopted and evaluation of MTP joints has increased in importance. Biz et al. [26] reported a favorable improvement in clinical scores and radiological evaluations of distal metatarsal metaphyseal osteotomy (DMMO), a percutaneous osteotomy without any fixation at the osteosynthesis materials on the osteotomy site. They described that all radiographic parameters of the Maestro criteria were significantly different at postoperative evaluation from the preoperative period. They concluded that Maestro criteria were useful to calculate which metatarsal bone should be shortened. On the other hand, they also showed that Maestro criteria do not have a predictive value in clinical outcomes of DMMO. They suggested that additional studies are needed to find more specific radiological measurements for preoperative planning of DMMO.

### 4.2. Classification of Lesser Toe Deformities

Mann et al. [27] reported a classification system that assesses existing dorsal dislocation of the MTP joint based on physical findings. The classification or grading of MTP joint deformities using plain radiograph lateral views has also been reported [28]. However, it is not easy to evaluate the lesser toes, especially the 3rd–5th toes, because these toes and metatarsal bone overlap in plain radiograph lateral views. Doorn et al. [29] graded the severity of RA forefoot deformities by evaluating clinical and radiographic factors, including the MTP joint ROM, destruction of the MTP joint on plain radiographs, soft tissues under the metatarsal head, and hallux valgus > 20°. Hirao et al. [30] also reported a classification scheme for second MTP joints by evaluating joint space narrowing, lateral shifting of the proximal phalanx, overlapping of the proximal phalanx base and the metatarsal head, and complete dorsal dislocation on plain radiographs. Of note, all of these gradings are qualitative assessments and not applicable for quantitative analysis of the severity or treatment results. The usefulness of the MOD lies in the simplicity of the measurement and quantification of each toe deformity.

Furthermore, the MOD grade was shown to be highly reproducible and significant correlation with the presence of plantar callosities. The percentage of toes with plantar callosities increased as the MOD grade increased. The shortening length of metatarsal bones required to correct MTP joint deformities was more extensive in cases with larger MODs. Therefore, the MOD appears to be a promising concept to assess the effectiveness of surgical interventions based on plain radiographs. This feature may assist the decision-making of surgical intervention, as well as preoperative planning. In our perspective, RA lateral toe deformity with MOD grade 2 is at the risk of developing forefoot pain or callosities, should be carefully followed to decide the indication of orthotic treatment or surgery. MOD grade 3 may indicate the formation of callosities and may support the indication of surgical interventions such as metatarsal osteotomy. For the preoperative planning and intraoperative decisions, more than half of the MOD thought to be adequate as the shortening length of metatarsal bones.

### 4.3. Shortening Oblique Osteotomies for Lesser Toes

**Figure 8:**
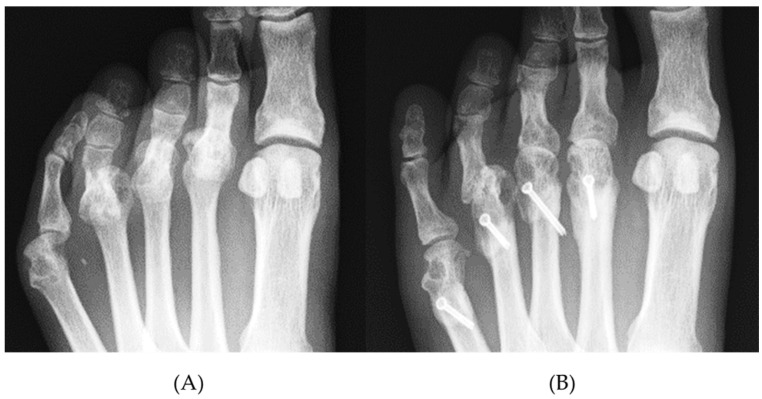
Radiographic findings before (**A**) and after (**B**) shortening oblique osteotomy for lesser metatarsal bones. The preoperative MOD of each toe was 15.9 mm (grade 3), 18.9 mm (grade 3), 17.4 mm (grade 3), and 5.5 mm (grade 2) in the 2nd–5th toes, respectively. Postoperative MOD grades were ameliorated to grade 1 in the 2nd, 3rd, 5th MTP joint. In the 4th MTP joint, postoperative MOD was 10.9 mm (grade 3), and there was a recurrent dorsal dislocation, causing the remnant callosity on the plantar side. The ratio of metatarsal shortening/preoperative MOD (%) was 30%.

Various methods of shortening osteotomies for lesser toes have been reported. In addition to the optimal osteotomy and fixation procedure, adequate shortening of metatarsal bones and ideal alignment of metatarsal heads have been discussed. Okuda et al. [31] assessed the results of metatarsal shortening osteotomy and reported that the top of metatarsal heads was 8–9 mm higher than the line drawn through the tops of the 1st and 5th metatarsal heads on postoperative radiographs, and reported that it was slightly less than the normal mean. Hanyu et al. [32] showed that the shortening was an average of 7 mm with an oblique osteotomy of metatarsal bones in a series involving 86 feet. They concluded that the postoperative lengths of the 2nd–5th metatarsal bones were longest in the 2nd metatarsal bone and shortened in the 3rd, 4th, and 5th metatarsal bones. Niki et al. [18,33] described a procedure in which the 1st metatarsal bones are aligned at nearly the same level as the 2nd metatarsal bone, which is shortened first. Subsequently, the heads of the 3rd and 4th metatarsal bones are sequentially shortened toward the lateral side to form a smooth arc [18,33]; however, the severity of each MTP joint deformity affects the shortening length of the metatarsal bones. Specifically, severe MTP joint dislocation causes soft tissue contracture and results in massive metatarsal bone shortening. Hirao et al. [13] described planning of metatarsal shortening length for each lesser toe based on the distance of overlap between the metatarsal head and the proximal phalanx. In the present study, we also investigated the shortening length at the osteotomy site. In all of the toes with postoperative remnant plantar callus, the metatarsal shortening length was <50% of the preoperative MOD value (Figure 8). This result indicated that the MOD value is a critical factor when discussing the amount of metatarsal shortening; however, no significant relationship was demonstrated between these factors and clinical assessment methods other than the Deformity subscale (JSSF, SAFEQ). Furthermore, we could not assess the MOD cut-off value for remnant plantar callosities because of the limited number of study samples, especially cases with postoperative remnant plantar calluses.

### 4.4. Limitations

The current study has some limitations. First, the specific conditions for radiographic examinations could affect the MOD measurement. Foot radiograph measurements significantly depend on the examination techniques, such as the patient foot position and the angle or distance of the X-ray tube [34]. Few studies clearly describe the imaging conditions used in reports involving the foot. In this study, the inclination of the X-ray tube was set to 30 degrees from the vertical axis in the sagittal plane, which did not prevent the RA patient from maintaining a standing position. With respect to the definition of the metatarsal axis, various methods have also been reported, majorly as an evaluation of the hallux valgus [35]. Shima et al. [22] reported that significantly different results were observed in five measurement methods with respect to intermetatarsal angles. They concluded that the method in which the longitudinal axis corresponds with the line connecting the center of the first metatarsal head and the center of the proximal articular surface of the metatarsal bone had the best intra- and inter-observer measurement reliability and agreement for the intermetatarsal angle measurements in patients managed with a proximal crescentic osteotomy of the first metatarsal bone. Second, the effects of hallux valgus and the first ray have not been considered in the analysis of postoperative callosity formation and clinical scores. Changes in the length and alignment of the first metatarsal alter the maestro line, causing an imbalance in weight-load distribution and mechanical overload to the affected metatarsal heads, which can cause the transfer of metatarsalgia and plantar callosities [36]. In the case of hypermobility of the first ray, alterations of normal foot mechanics are present. The vertical reaction force to the ground raises the first metatarsal head over the plane of the lesser metatarsal heads, moving the load onto the lesser metatarsals. This can lead to a collapse of the internal longitudinal arch of the foot [37] and may affect the result of metatarsal osteotomies. Third, as described in the current study, the optimal cut-off values of MOD differed from each toe. The different trends in MTP joint deformities for each toe, such as dorsal dislocation or varus deformity, were not considered in this analysis. For example, the AUC value calculated in the ROC analysis for the 5th toe was relatively low, suggesting that other factors affect the 5th toe plantar callosity formation more strongly than others (e.g., mid- to hindfoot deformities).

In addition, the cut-off values may differ according to foot morphology, physique, and race. A prospective investigation with a large population is warranted.

## 5. Conclusions

In conclusion, the findings of this study suggest that MOD, as measured on plain radiographs, is useful for evaluating MTP joint deformities involving the lesser toe in patients with RA. The MOD and MOD grade were significantly correlated with the presence of plantar callosities and reflected the results of joint preservation surgery of the lesser toe. Therefore, this novel measurement method might contribute to decide or discuss the indications for surgical intervention, risk factors for recurrence, and the adequate shortening length of metatarsal bones.

## Figures and Tables

**Table 1:** The mean MOD of toes with or without callosities.

	with Callosities		without Callosities				
	Number of Toes	MOD (mm)	Number of Toes	MOD (mm)	*p* Value	AUC	Cut-Off Value (mm)
2nd	63	14.0 ± 8.1	213	3.2 ± 3.8	<0.001	0.90	10
3rd	66	14.7 ± 7.1	210	3.4 ± 3.1	<0.001	0.91	8.4
4th	20	16.8 ± 4.7	256	4.0 ± 4.9	<0.001	0.95	9.8
5th	25	8.5 ± 4.4	251	4.1 ± 2.7	<0.001	0.80	6.6
Total	174	14.0 ± 7.3	930	3.7 ± 3.8	<0.001	0.89	7.8

MOD: metatarsophalangeal overlap distance. MODs are expressed as the mean ± SD. Statistical significance was calculated using Student’s *t*-test. AUC: area under the curve.

**Table 2:** Pre- and post-operative MODs, MOD grades, and clinical parameters.

		Preoperative	Postoperative	*p* Value
MOD (mm)		10.7 ± 7.5	4.8 ± 2.4	<0.001
MOD grade	grade 1	31	75	<0.001
(toes)	grade 2	28	39	
	grade 3	57	2	
JSSF scale	General pain	21.0 ± 3.1	28.1 ± 4.0	<0.001
	Deformity	15.1 ± 3.5	21.3 ± 2.7	<0.001
	Motion	9.4 ± 3.6	11.0 ± 2.6	0.006
	Walking ability	12.8 ± 4.5	18.9 ± 3.2	<0.001
	ADL	4.0 ± 2.2	5.6 ± 2.8	<0.001
	Total	62.4 ± 7.5	85.0 ± 9.4	<0.001
SAFE-Q	Pain-related	59.3 ± 17.1	68.0 ± 14.5	0.016
	Physical Functioning and Daily Living	65.6 ± 17.2	71.0 ± 14.8	0.005
	Social Functioning	70.0 ± 23.1	76.6 ± 17.5	0.021
	Shoe-related	44.3 ± 21.3	51.9 ± 27.0	0.062
	General Health and Well-being	65.7 ± 24.5	75.2 ± 23.2	0.002

MODs are expressed as the mean ± SD. Each value was compared using a paired *t*-test or chi-square test. JSSF: Japanese Society of Surgery of the Foot, ADL: activities of daily living, SAFE-Q; self-administered foot evaluation questionnaire.

**Table 3:** Correlation between Δ MOD and Δ clinical scores.

		r Value	*p* Value
Δ JSSF scale	General pain	0.24	0.22
	Deformity	0.46	0.02
	Motion	0.08	0.68
	Walking ability	−0.24	0.23
	ADL	0.25	0.22
	Total	0.29	0.15
Δ SAFE-Q	Pain-related	−0.11	0.59
	Physical Functioning and Daily Living	−0.14	0.51
	Social Functioning	−0.33	0.10
	Shoe-related	0.08	0.69
	General Health and Well-being	−0.20	0.33

Statistical significance was calculated by Pearson’s correlation.

## Data Availability

The data that support the findings of this study are available from the corresponding author, Keiichiro Nishida, upon reasonable request.
